# Blood Glucose Abnormalities and Their Association With Mortality Among Neonates With Sepsis: A Prospective Observational Study From a Tertiary Care Center in Central India

**DOI:** 10.7759/cureus.114603

**Published:** 2026-08-16

**Authors:** Vrushank Patel, Saurabh Piparsania, Stuti Gagrani

**Affiliations:** 1 Pediatrics, Index Medical College, Hospital & Research Centre, Indore, IND

**Keywords:** blood glucose, neonatal hyperglycemia, neonatal hypoglycemia, neonatal intensive care unit (nicu), neonatal mortality, neonatal sepsis

## Abstract

Background: Neonatal sepsis remains a leading cause of neonatal morbidity and mortality, particularly in resource-limited settings. Beyond infection itself, metabolic disturbances, such as abnormalities of blood glucose, may influence disease severity and outcome. Data on the prognostic significance of glycemic derangement in septic neonates from Indian tertiary care units remain limited.

Objective: This study aimed to determine the prevalence of blood glucose abnormalities among neonates with sepsis and to assess their association with in-hospital mortality.

Materials and methods: This prospective observational study was conducted over 18 months in the level III NICU of Index Medical College Hospital and Research Centre, Indore, a tertiary care referral center, and included 100 consecutively enrolled neonates with proven, probable, or possible sepsis. Blood glucose was measured within one hour of admission and categorized as hypoglycemia (<40 mg/dL), normoglycemia (40-145 mg/dL), or hyperglycemia (>145 mg/dL). Clinical features, laboratory parameters, microbiological findings, and outcomes were recorded on a predesigned proforma. Analysis was performed using IBM SPSS Statistics version 26.0 (IBM Corp., Armonk, NY, USA); a two-sided p-value <0.05 was considered statistically significant.

Results: Early-onset sepsis predominated (88/100, 88.0%). Glycemic abnormalities were present in 28/100 (28.0%) neonates; 17/100 (17.0%) had hypoglycemia, and 11/100 (11.0%) had hyperglycemia. Overall mortality was 11/100 (11.0%). Mortality was significantly higher among hypoglycemic (5/17, 29.4%) and hyperglycemic (3/11, 27.3%) neonates than among normoglycemic neonates (3/72, 4.2%). Respiratory distress was the commonest presentation (48/100, 48.0%), and blood cultures were positive in 18/100 (18.0%) neonates, with *Staphylococcus aureus* isolated in 9/100 (9.0%).

Conclusions: Glycemic derangements are common in neonatal sepsis and are significantly associated with increased in-hospital mortality. Blood glucose measurement at admission is inexpensive and universally available and may serve as a simple bedside prognostic indicator requiring validation in larger multicenter studies.

## Introduction

Neonatal sepsis is a major cause of neonatal morbidity and mortality worldwide, particularly in low- and middle-income countries. It is defined as a systemic infection occurring within the first 28 days of life and remains a critical challenge in neonatal care despite advances in medical management [1,2]. An estimated three million neonates develop sepsis each year globally, and India contributes a substantial proportion of these cases and of the resulting mortality, reflecting constrained resources, delayed diagnosis, and the heightened susceptibility of preterm and low-birth-weight infants [3,4].

Neonatal sepsis is classified as early-onset or late-onset on the basis of the timing of presentation and the presumed mode of transmission. Clinical manifestations are frequently nonspecific and include respiratory distress, poor feeding, lethargy, temperature instability, and multiorgan dysfunction, which makes early diagnosis difficult [5]. These infections trigger systemic inflammatory responses and metabolic alterations that substantially affect neonatal physiology.

Glucose is the principal energy substrate for the neonatal brain, and maintenance of normal glucose concentrations is essential for survival and neurological development. Sepsis disrupts glucose homeostasis through increased metabolic demand, hormonal imbalance, and release of inflammatory cytokines, producing either hypoglycemia or hyperglycemia [6,7]. Hypoglycemia may cause neuronal injury and adverse neurodevelopmental outcomes, whereas hyperglycemia has been associated with increased mortality and impaired immune function [8,9].

Monitoring blood glucose in septic neonates is therefore essential for the early detection of metabolic disturbance. Clarifying the relationship between glycemic status at admission and clinical outcome may improve prognostication and guide management. Although previous studies have demonstrated an association between dysglycemia and adverse neonatal outcomes, prospective evidence from Indian tertiary-care neonatal intensive care units remains limited [9,10]. The present study was undertaken to determine the prevalence of blood glucose abnormalities among neonates with sepsis admitted to a level III NICU and to assess their association with in-hospital mortality.

## Materials and methods

Study design and setting

This prospective observational study was conducted in the level III NICU of Index Medical College Hospital and Research Centre, Indore, a tertiary care referral center providing advanced neonatal services to both inborn and outborn neonates. The study ran for 18 months, from April 1, 2024, to September 30, 2025. Standard treatment protocols were not altered for the purposes of the study.

Participants and sample size

A total of 100 neonates with clinically suspected or proven sepsis who were admitted to the NICU during the study period were included in the study. Participants were enrolled consecutively until the predetermined sample size was achieved. Neonates with proven sepsis (positive blood culture with compatible clinical features), probable sepsis (clinical features with a positive sepsis screen and negative blood culture), or possible sepsis (clinical features with at least one recognized maternal or perinatal risk factor for sepsis) were eligible for inclusion. Because blood culture positivity is often limited in neonatal sepsis, neonates with probable and possible sepsis were also included to capture the spectrum of clinically suspected sepsis encountered in routine NICU practice. Accordingly, the study population comprised neonates with different levels of diagnostic certainty, and the findings were interpreted as applying to clinically suspected or proven sepsis rather than to culture-confirmed sepsis alone.

The maternal and perinatal risk factors documented in the study included prematurity and leaking per vaginum. Maternal parity was also recorded. The presence of at least one of these risk factors in association with compatible clinical features was considered sufficient for classification as possible sepsis. The sepsis screen comprised total leukocyte count (TLC), absolute neutrophil count (ANC), immature-to-total neutrophil (I:T) ratio, C-reactive protein (CRP), and micro-erythrocyte sedimentation rate (micro-ESR). The screen was considered positive when at least two parameters were abnormal: TLC <5,000/mm³, ANC <1,800/mm³ or below the age-specific reference range, I:T ratio ≥0.20, CRP ≥10 mg/L (1 mg/dL), or micro-ESR >15 mm in the first hour. Preterm (<37 weeks), term (37-42 weeks), and post-term (>42 weeks) neonates were eligible for inclusion. Neonates were excluded if parental consent was not obtained, blood glucose measurement could not be performed within one hour of admission because of the need for immediate stabilization or resuscitation, or they were discharged against medical advice.

For the purpose of this study, early-onset sepsis was operationally defined as sepsis presenting within the first five days of life, whereas sepsis presenting after five days was considered late-onset sepsis.

The sample size was calculated using the formula for estimating a single population proportion:

n = (Z² × p × q) / d²

where *n* is the required sample size, *Z* is the standard normal deviate corresponding to a 95% confidence level (1.96), *p* is the anticipated prevalence of blood glucose abnormalities among neonates with sepsis, *q = 1 − p*, and *d* is the absolute precision.

Based on a study conducted in Egypt [11], which reported a prevalence of 23.6% of blood glucose abnormalities among neonates with sepsis, the anticipated prevalence (p) was taken as 0.236. Using a 95% confidence level (Z = 1.96) and an absolute precision of 9% (d = 0.09), the minimum required sample size was calculated as 86 neonates, which was increased to 100 neonates as the predetermined final sample size. Consecutive eligible neonates were enrolled until the required sample size was achieved.

Study procedures

After written informed consent was obtained, a detailed clinical history including antenatal, intrapartum, and postnatal details was recorded. A complete physical examination was performed, with particular attention to vital parameters and clinical features suggestive of sepsis. Venous blood was collected under strict aseptic precautions for complete blood count, C-reactive protein (CRP), band cell count, toxic granules, absolute neutrophil count, and blood culture. All samples were transported to the laboratory within 30 minutes of collection. All neonates were managed according to standard NICU protocols.

Blood glucose measurement

Blood glucose was measured within one hour of admission for suspected or proven sepsis using a Gluco Navii glucometer (Diatech Diabetescare, Gujarat, India) based on the glucose oxidase method. For neonates who developed clinical features of sepsis after admission to the NICU, the blood glucose measurement obtained within one hour of recognition of suspected sepsis and initiation of sepsis evaluation was considered the admission blood glucose value. Thus, glucose measured at the time of birth or at the initial NICU admission before development of sepsis was not considered the study admission glucose value. Heel-prick samples were used, and plasma-equivalent glucose values were recorded. Subsequent glucose monitoring followed unit protocol. On the basis of the admission value, neonates were categorized as hypoglycemic (<40 mg/dL), normoglycemic (40-145 mg/dL), or hyperglycemic (>145 mg/dL). The <40 mg/dL cutoff was applied to the glucose measurement obtained during sepsis evaluation and was not intended to classify routine transitional glucose values immediately after birth.

Blood culture

Blood culture was performed in all neonates with suspected sepsis. Approximately 2-3 mL of venous blood was collected under aseptic precautions, inoculated into brain-heart infusion broth culture bottles, and incubated. Culture and sensitivity reports were available after 48-72 hours.

Outcome measures

The primary outcome was the prevalence of glycemic abnormalities at admission. The secondary outcome was in-hospital mortality and its association with admission glycemic status.

Statistical analysis

Data were entered on a predesigned structured proforma and analyzed using IBM SPSS Statistics version 26.0 (IBM Corp., Armonk, NY, USA). Quantitative variables were expressed as mean ± standard deviation and qualitative variables as frequency and percentage. Fisher's exact test was used whenever the expected cell frequency was less than five. Associations between categorical variables were assessed using Pearson's chi-square test. A two-sided p-value <0.05 was considered statistically significant.

Ethical approval

The study protocol was reviewed and approved by the Institutional Ethics Committee of Index Medical College Hospital and Research Centre, Indore, Madhya Pradesh, India (IEC Reference No. IMCHRC/IEC/2024/87; approval dated 20 April 2024; IEC Registration No. ECR/708/Inst/MP/2015). The study was conducted in accordance with the ethical principles of the Declaration of Helsinki. Written informed consent was obtained from the parents or legally authorized guardians of all enrolled neonates before participation.

## Results

A total of 100 neonates with clinically suspected or proven sepsis were included. Most neonates presented within the first five days of life (88/100, 88.0%), indicating a predominance of early-onset sepsis. There was a slight female predominance (51/100, 51.0%). Most neonates were delivered by lower-segment cesarean section (60/100, 60.0%), and 62/100 (62.0%) were born at a gestational age >37 weeks. Low birth weight (≤2.5 kg) was present in 58/100 (58.0%) neonates, while 83/100 (83.0%) were inborn. Prematurity was the commonest documented perinatal risk factor (26/100, 26.0%), followed by leaking per vaginum (16/100, 16.0%). Baseline maternal and neonatal characteristics are shown in Table 1.

**Table 1:** Baseline maternal and neonatal characteristics (N = 100) Percentages are calculated out of the total of 100 neonates. Categories are not mutually exclusive.

Variable	Number (n)	Percentage (%)
Age at presentation ≤5 days	88	88.0
Female sex	51	51.0
Delivery by lower segment cesarean section	60	60.0
Gestational age >37 weeks	62	62.0
Birth weight ≤2.5 kg	58	58.0
Inborn (intramural) birth	83	83.0
Nulliparous mother	37	37.0
Prematurity as maternal risk factor	26	26.0
Leaking per vaginum as maternal risk factor	16	16.0

Among the clinical manifestations recorded in the study, respiratory distress was the commonest presentation (48/100, 48.0%), followed by feed intolerance (22/100, 22.0%) and temperature instability (12/100, 12.0%). Toxic granules were present in 38/100 (38.0%) neonates, while blood cultures were positive in 18/100 (18.0%). Clinical and laboratory characteristics are summarized in Table 2.

**Table 2:** Clinical and laboratory characteristics (N = 100) Percentages are calculated out of the total of 100 neonates. Sepsis classification categories are mutually exclusive; clinical features are not mutually exclusive.

Variable	Number (n)	Percentage (%)
Sepsis classification		
Proven sepsis	18	18.0
Probable sepsis	47	47.0
Possible sepsis	35	35.0
Clinical characteristics		
Respiratory distress	48	48.0
Feed intolerance	22	22.0
Temperature instability	12	12.0
Laboratory characteristics		
Toxic granules present on peripheral smear	38	38.0
Blood culture positive	18	18.0

The mean hemoglobin was 15.56 ± 2.73 g/dL, mean CRP was 31.68 ± 50.83 mg/L, mean total leukocyte count was 17,121 ± 9,474/µL, and mean platelet count was 114.28 ± 57.79 × 10⁹/L (Table 3). The wide standard deviation for CRP reflects a considerable spread of inflammatory response across the cohort.

**Table 3:** Hematological and inflammatory parameters (N = 100) SD: standard deviation

Parameter	Mean ± SD	Unit
Hemoglobin	15.56 ± 2.73	g/dL
C-reactive protein	31.68 ± 50.83	mg/L
Total leukocyte count	17,121 ± 9,474	/µL
Platelet count	114.28 ± 57.79	× 10⁹/L

Blood cultures were positive in 18/100 (18.0%) neonates. *Staphylococcus aureus* was the commonest isolate, accounting for 9/18 (50.0%) culture-positive isolates and 9/100 (9.0%) of the overall cohort. The distribution of isolated organisms is shown in Table 4.

**Table 4:** Distribution of organisms isolated on blood culture (N = 100)

Organism	Number (n)	% of total cohort (N = 100)	% of culture-positive isolates (n = 18)
Staphylococcus aureus	9	9.0	50.0
Klebsiella species	3	3.0	16.7
Pseudomonas species	3	3.0	16.7
Escherichia coli	2	2.0	11.1
Citrobacter species	1	1.0	5.6
Total culture-positive	18	18.0	100.0
Culture-negative	82	82.0	0.0

Glycemic status

**Figure 1:**
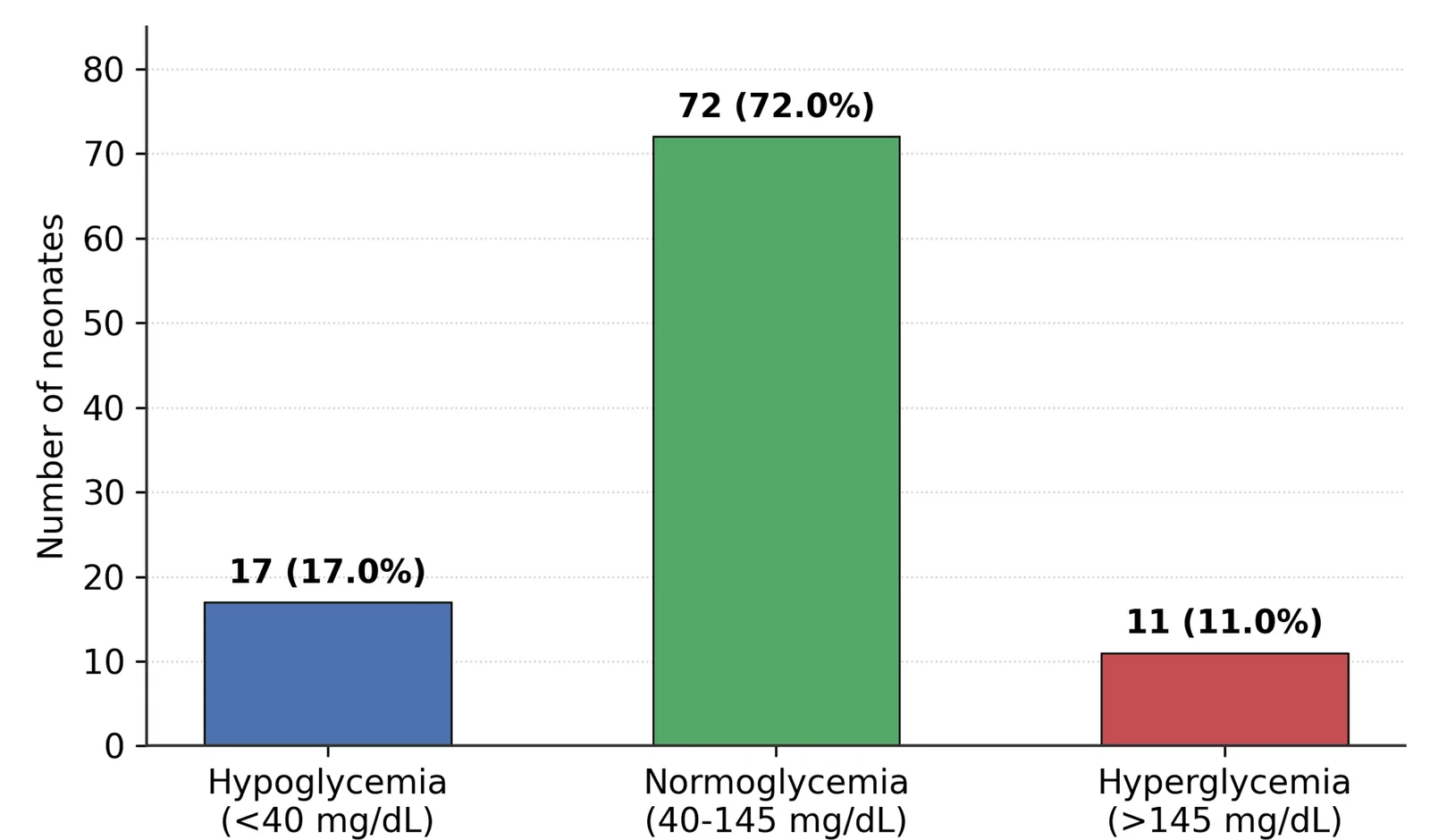
Distribution of glycemic status at admission among neonates with sepsis (N = 100)

Seventy-two (72/100; 72.0%) neonates were normoglycemic at admission, whereas 17/100 (17.0%) had hypoglycemia and 11/100 (11.0%) had hyperglycemia, giving an overall prevalence of glycemic abnormality of 28/100 (28.0%) (Figure 1).

Glycemic status and mortality

**Figure 2:**
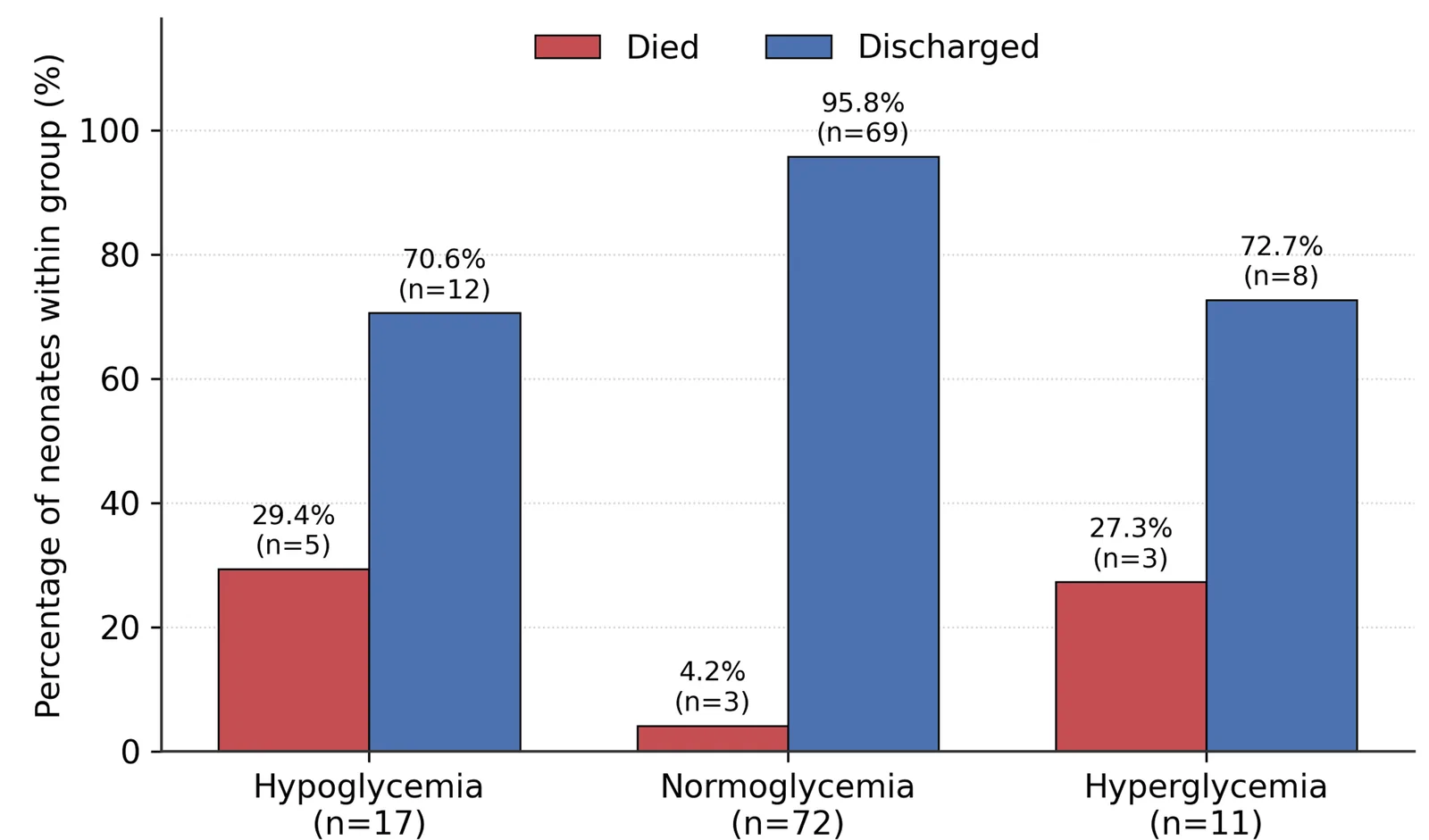
In-hospital outcome by glycemic status at admission among neonates with sepsis (N = 100) Bars represent the percentage of neonates within each glycemic category; absolute numbers are shown above each bar. Pearson's chi-square = 12.296, df = 2, p = 0.002.

Overall in-hospital mortality was 11/100 (11.0%). Mortality occurred in 5/17 (29.4%) hypoglycemic neonates, 3/11 (27.3%) hyperglycemic neonates, and 3/72 (4.2%) normoglycemic neonates. The mortality rate among neonates with any glycemic abnormality (8/28, 28.6%) was substantially higher than among normoglycemic neonates (3/72, 4.2%). The association between glycemic status and outcome was statistically significant (χ² = 12.296, df = 2, p = 0.002) (Figure 2).

## Discussion

Neonatal sepsis remains a leading cause of neonatal morbidity and mortality worldwide, particularly in low- and middle-income countries [1,3]. Beyond infection itself, systemic metabolic disturbance materially influences disease severity and clinical outcome, and abnormalities of glucose homeostasis are increasingly recognized as early markers of physiological stress and predictors of adverse outcomes [9,11]. The present study evaluated the demographic profile, clinical presentation, laboratory parameters, microbiological spectrum, glycemic status, and outcomes of septic neonates, with particular emphasis on the prognostic relevance of blood glucose abnormality.

Timing of onset and demographic characteristics

In the present study, 88.0% of neonates presented within the first five days of life, indicating a predominance of early-onset neonatal sepsis. Comparable findings have been reported from developing countries, where early-onset sepsis accounts for the majority of NICU admissions for infection [1,4]. Early-onset sepsis is commonly associated with vertical transmission and perinatal stress, both of which may predispose neonates to metabolic instability, including impaired glucose regulation.

The nearly equal sex distribution observed here differs from reports suggesting male predominance in neonatal sepsis, an observation often attributed to X-linked immune modulation [12]. Variable or near-equal sex distributions have, however, also been described, suggesting that environmental and health-system factors may outweigh biological predisposition in particular populations.

Perinatal and maternal risk factors

Prematurity was the commonest documented perinatal risk factor identified. Preterm neonates have limited glycogen reserves, immature hepatic gluconeogenic pathways, and blunted counter-regulatory hormonal responses, which render them highly susceptible to hypoglycemia during systemic illness [6,8]. Leaking per vaginum, recorded in 16.0% of mothers, reinforces the role of ascending infection in early-onset sepsis [1].

Gestational age and birth weight

Although 62.0% of neonates in this cohort were born at more than 37 weeks of gestation, low birth weight was nonetheless present in 58.0%, indicating a substantial burden of fetal growth restriction in addition to prematurity. Low birth weight neonates are particularly prone to hypoglycemia because of reduced glycogen stores, diminished adipose tissue, and increased metabolic demand during infection [8]. The coexistence of term gestation with low birth weight in a large share of this cohort is itself noteworthy and may reflect the referral profile of the center.

Clinical manifestations

Respiratory distress was the commonest presenting feature, followed by feed intolerance and temperature instability. These findings are consistent with those of Shane et al. and Polin, in which respiratory and feeding difficulties were early indicators of systemic infection [1,5]. Hypoglycemia has been shown to contribute to neurological depression and impaired cardiovascular function in critically ill neonates [6].

Hematological and inflammatory parameters

The elevated CRP concentrations, leukocytosis, thrombocytopenia, and presence of toxic granules observed in this study reflect the systemic inflammatory response characteristic of neonatal sepsis [1,13]. Thrombocytopenia in particular has been associated with severe sepsis and increased mortality [13]. These findings indicate that glycemic abnormalities occurred in a clinical setting characterized by hematological and inflammatory abnormalities; however, this study did not specifically evaluate the correlation or causal relationship between glycemic status and these parameters.

Microbiological profile

The culture positivity rate of 18.0% observed here is consistent with rates reported from comparable Indian settings. The predominance of *Staphylococcus aureus *aligns with Indian NICU-based data in which gram-positive organisms are frequently isolated [10]. The high proportion of culture-negative sepsis underscores the limitations of microbiological confirmation in this setting and the value of adjunctive markers, including glycemic status, for early risk stratification [13].

Prevalence of glycemic abnormality

In the present study, 28.0% of neonates had an abnormal admission blood glucose. Similar prevalence figures have been reported in neonatal sepsis cohorts, in which glucose abnormalities have ranged from approximately 20% to 40% [10,14]. Hypoglycemia in sepsis may result from increased glucose consumption, impaired gluconeogenesis, hepatic dysfunction, and adrenal insufficiency [6,15]. Conversely, hyperglycemia reflects stress-induced insulin resistance mediated by inflammatory cytokines and counter-regulatory hormones [7,15]. Both abnormalities indicate severe physiological stress and impaired metabolic adaptation.

Glycemic status and mortality

A principal finding of the present study was the statistically significant association between glycemic abnormality and mortality. Mortality was substantially higher in neonates with either hypoglycemia or hyperglycemia than in normoglycemic neonates. These findings support the concept of a U-shaped relationship between blood glucose concentration and mortality in critically ill patients [9,16]. Similar observations have been reported in neonatal populations, in which both extremes of glycemia predicted adverse outcomes [11], and hypoglycemia has been linked specifically to early-onset sepsis in premature infants [14]. However, glycemic status in the present study was based on a single admission glucose measurement. Serial glucose measurements may provide a more comprehensive assessment of persistent or fluctuating dysglycemia and may better characterize its relationship with clinical deterioration and mortality. Therefore, the prognostic significance of a single admission glucose value should be interpreted cautiously, and future prospective studies incorporating serial glucose measurements and glycemic variability are warranted.

The observed association should also be interpreted in the context of other factors that may influence neonatal glycemic status, including prematurity, small- or large-for-gestational-age status, and being an infant of a diabetic mother [17]. Low birth weight and severity of illness may also influence glycemic status and mortality. These factors were not comprehensively assessed or adjusted for in the present study and may have contributed to the observed association. In addition, 88.0% of neonates presented within the first five days of life, consistent with the study's operational definition of early-onset sepsis.

It should be emphasized that this association does not establish causation. Glycemic abnormalities may reflect underlying illness severity rather than represent an independent predictor of mortality. Furthermore, the present analysis was unadjusted for gestational age, birth weight, and severity of illness, all of which are plausible confounders. These findings support the routine assessment of admission blood glucose as part of the initial evaluation of neonates with suspected sepsis, particularly in resource-limited settings where rapid and inexpensive prognostic indicators are valuable.

Clinical implications

The present study reinforces accumulating evidence that glycemic derangement is common in neonatal sepsis and is associated with increased in-hospital mortality. Blood glucose measurement is simple, inexpensive, and widely available, including in units without access to advanced biomarkers. Early identification of glycemic abnormalities may facilitate prompt risk stratification, timely clinical assessment, and appropriate supportive management.

Limitations

This study has several limitations. This was a single-center study with a modest sample size, which limits generalizability and precluded multivariable adjustment for potential confounders such as gestational age, birth weight, small- or large-for-gestational-age status, maternal diabetes, and severity of illness. Blood glucose was classified on the basis of a single point-of-care measurement at admission; glucometer readings are known to be less accurate than laboratory plasma glucose at the extremes of the range, and serial values, glycemic variability, and the effect of glucose-directed therapy were not captured. The subgroups of hypoglycemic and hyperglycemic neonates were small (17 and 11 neonates, respectively), so the mortality estimates within these strata carry wide confidence intervals. Sepsis was defined clinically in the majority of cases, since 82.0% of blood cultures were negative. Although this reflects the clinical reality of neonatal sepsis, inclusion of proven, probable, and possible sepsis resulted in different levels of diagnostic certainty and may have introduced some risk of misclassification. Neurodevelopmental follow-up after discharge was not undertaken.

## Conclusions

Glycemic abnormalities were common in neonates with sepsis and were significantly associated with higher in-hospital mortality. Routine blood glucose estimation at admission may provide a simple and inexpensive bedside marker associated with increased mortality and may assist in early risk stratification. Larger multicenter studies with multivariable adjustment are required to validate these findings.
